# Regulatory roles of noncoding RNAs in oil palm response to cold stress

**DOI:** 10.3389/fpls.2025.1685230

**Published:** 2025-10-22

**Authors:** Qiufei Wu, Rui Li, Xianhai Zeng, Dengqiang Fu, Qihong Li, Zongming Li, Hongxing Cao, Xinyu Li, Xiaoyu Liu, Lixia Zhou

**Affiliations:** State Key Laboratory of Tropical Crop Breeding, Coconut Research Institute, Chinese Academy of Tropical Agricultural Sciences, Sanya, Hainan, China

**Keywords:** oil palm, cold stress, transcriptome, lncRNA, microRNA, palm oil synthesis

## Abstract

**Introduction:**

Noncoding RNAs (ncRNAs) are crucial regulators of cellular functions and are actively expressed in different tissues and throughout various stages of development. However, their roles in oil palm (*Elaeis guineensis* Jacq.) under abiotic stress, particularly cold stress, remain poorly understood.

**Methods:**

We profiled spear leaves across a cold-stress time course (0–8 h at 8°C), and conducted an in-depth transcriptome analysis to explore and characterize differentially expressed genes (DEGs), differentially expressed microRNAs (DEMs), and differentially expressed lncRNAs (DELs) in oil palm subjected to cold stress, aiming to elucidate the regulatory networks among these molecules. We called DE with |log2FC|≥1 (DEGs/DELs: FDR<0.05; DEMs: p<0.05).

**Results and discussion:**

Comparative analysis revealed 1,106 DELs, 638 DEMs and 13,539 DEGs reacting to cold stress relative to control conditions (CK). GO and KEGG enrichment of DEGs and predicted ncRNA targets highlighted carbohydrate/lipid metabolism and secondary-metabolite biosynthesis. Furthermore, the study demonstrated that miR156-zmiR156-z negatively regulated *FabF1* in protoplasts, providing targeted functional validation within the inferred network. The findings offer new perspectives on the regulatory role of ncRNAs in oil palm’s response to cold stress and establish a basis for future functional research. Gaining insight into these molecular mechanisms may help improve cold resilience in oil palm, paving the way for the development of more robust cultivars.

## 1 Introduction

Cold stress is a significant environmental factor that impacts vegetative growth, development, crop yield and geographic occurrence (Naing and Kim, 2021; Kopecká et al., 2023). Oil palm, originating from West Africa and belonging to the Arecaceae family, is mainly grown for its oil production (Cros et al., 2015). As a typical tropical oil crop, oil palm thrives best within a temperature range of 25-27°C for optimal growth. Cold stress can significantly threaten oil palm productivity, resulting in substantial economic implication (Qaim et al., 2020). Therefore, it is crucial to develop oil palm varieties that can adapt to changing environmental conditions through molecular breeding approaches. So far, only a small number of genes involved in responses to abiotic stress have been recognized in the oil palm genome (Xiao et al., 2017; Zhou and Yarra, 2021; Zhou et al., 2020). Further, advancement in understanding the expression patterns of these important functional genes can be achieved by studying the complex regulatory networks involving messenger RNAs (mRNAs), micro RNAs (miRNAs), and long noncoding RNAs (lncRNAs).

miRNAs are short, ncRNAs present in eukaryotes, characterized by their origin from single stranded RNA precursors that fold into a hairpin structure. These molecules usually range from 20–24 nucleotides in length and performs a central task in regulating gene expression in plants. A groundbreaking finding demonstrated that miRNAs can modulate cell development in nematodes (*Caenorhabditis elegans*), laying the foundation for future research on miRNAs (Lee et al., 1993). As research has progressed, our knowledge of miRNAs has expanded, leading to the identification of numerous miRNAs and a deeper understanding of their biosynthesis and mechanisms. miRNAs contribute significantly to various biological processes, including regulating gene expression (Bartel, 2004; Song et al., 2019), secondary growth (Karlova et al., 2014; Nguyen et al., 2015), lignin biosynthesis (Fan et al., 2020), and responding to biotic and abiotic stresses (Olejniczak et al., 2018; Shah and Ullah, 2021; Gao et al., 2022). Moreover, miR156, found in both vegetative and reproductive tissues of oil palms, could play a role in controlling the development of male and female inflorescences (Zhou and Yarra, 2023). On the other hand, lncRNAs are RNA molecules longer than 200 nucleotides (nt) that do not code for proteins, and advances in high-throughput sequencing have identified numerous lncRNAs in plant species (Liu et al., 2020; Xie et al., 2022). LncRNAs are core regulators in plants, involved in processes, like seed germination (Nonogaki, 2014; Wu et al., 2019), seedling development (Chen et al., 2020), vegetative growth (Liang et al., 2022), reproductive development, and stress responses (Ginckels and Holvoet, 2022; Wang et al., 2023). However, lncRNAs have been profiled only sparingly in oil palm (Xia et al., 2020; Xu et al., 2024), their roles in cold stress remain unknown.

LncRNA, miRNA and mRNA work together within regulatory networks, where 69 and 25 lncRNAs function as target mimics for miRNAs in rice subjected to deep water stress. Competing endogenous RNA (ceRNA) analyses showed that some miRNAs were upregulated, while their corresponding lncRNAs targets exhibited opposite expression patterns (Panda et al., 2022). LncRNAs, miRNAs, circRNAs and mRNAs of Baiye (*Camellia sinensis*) were characterized under cold stress, and ceRNA regulatory networks were developed to examine their involvement in the cold stress (Xu et al., 2023). High throughput sequencing allows for the identification and analysis of lncRNAs, miRNAs and mRNAs, aiding in the understanding of their roles in post transcriptional biological processes (Chan and Tay, 2018). Investigating the interactions between lncRNA, miRNA and mRNA under cold stress is essential for uncovering the mechanisms of cold tolerance in oil palm. Through RNA-seq and sRNA sequencing, we identified important differentially expressed genes, miRNAs, and lncRNAs, and developed interaction networks to better understand the molecular pathways involved in the cold stress response. These insights could contribute to the creation of oil palm varieties with improved cold tolerance. We hypothesized that cold triggers coordinated changes in miRNAs and lncRNAs that reprogram metabolic and stress-response pathways, and that specific interactions such as miR156-z targeting *FabF1* mediate lipid-metabolism adjustments. Our objectives were to: (i) profile DELs/DEMs/DEGs across a cold time course; (ii) infer cis/trans/eTM and miRNA-target networks; (iii) identify hubs; and (iv) validate the miR156-z–*FabF1* interaction.

## 2 Materials and methods

### 2.1 Plant material and stress treatment

African oil palm (*Elaeis guineensis* var. pisifera, thin-shelled) seedlings were obtained from Wenchang, Hainan Province, China (19.63°N, 110.94°E) and cultivated in nurseries prior to the application of stress treatments. A set of oil palm plants, totaling eighteen and germinated within the same week under uniform nursery conditions, was selected for the subsequent cold treatment. A 16 hours light and 8 hours dark photoperiod at 26°C was used for seedling cultivation, with three independent biological replicates included for each stress treatment. The experiment included three biological replicates per timepoint (0, 0.5, 1, 2, 4, and 8 hours at 8°C), with each replicate consisting of a single seedling; resulting in a total sample size (n) of 18. Spear leaves were collected from both CK and cold treated seedlings and promptly frozen in liquid nitrogen for subsequent RNA isolation.

### 2.2 sRNA profiling and miRNA identification in oil palm

RNA was isolated from each sample using the Trizol reagent kit (Invitrogen, Carlsbad, CA, USA), and the samples were subsequently treated with DNase I (TaKaRa, China) to remove contaminating genomic DNA. The total RNA concentration and integrity were assessed using a Nanodrop 2000 Spectrophotometer (Thermo Fisher, USA) and an Agilent 2100 Bioanalyzer (Agilent, USA).

RNA fragments ranging from 18 to 30 nt in length were isolated using polyacrylamide gel electrophoresis (PAGE). Next, 3′ adapters were ligated to the RNA, and fragments between 36–48 nt RNAs were selectively enriched. Subsequently, 5′ adapters were attached, and the RNA underwent reverse transcription followed by PCR amplification. PCR products ranging from 140–160 bp were selectively enriched to construct a cDNA library, which was then sequenced using the Illumina HiSeq Xten platform at Gene Denovo Biotechnology Co. (Guangzhou, China).

The sequencing reads contained unwanted sequences, such as adapters remnants or low quality bases, which could impact the subsequent analysis. To obtain high quality tags, the raw reads were processed to remove low quality sequences, adapter contaminants, ploy A sequences, and reads shorter than 18 nt (excluding adapters). The clean tags were compared to small RNAs sequences in the GeneBank database (Release 209.0) and the Rfam database (Release 11.0). This alignment helped identify and remove transfer RNA (tRNA), small nuclear RNA (snRNA), small nucleolar RNA (snoRNA), small cytoplasmic RNA (scRNA), and ribosomal RNA (rRNA). The remaining clean tags were mapped to the reference genome of oil palm (Singh et al., 2013). All clean tags were then queried against the miRBase database (Release 22) to identify known miRNAs (Griffiths-Jones et al., 2006). Additionally, they were compared with the PNRD database (Yi et al., 2015) to detect previously characterized miRNA. All unannotated tags were mapped to the reference genome. Based on their genomic locations and the hairpin structures predicted by the Mirdeep2 software, potential novel miRNA were recognized. The parameter configuration for miRDeep2 was set as follows: -e -18 -d 300 -p 16 -v 4 -s 4 -f 20, with each parameter integrated into the analysis pipeline as described below: (1) Score threshold: Candidate miRNAs with a miRDeep2 score greater than 4 (as specified by the parameter -s 4) were retained for further analysis. (2) Energy threshold: The minimum free energy (MFE) of the predicted precursor miRNA hairpin structure was required to be ≤ -18 kcal/mol (as defined by the parameter -e -18). (3) Hairpin structure criteria: The built-in algorithm of miRDeep2 was used to assess whether candidate sequences could form typical hairpin structures. Parameter settings such as -v 4 (which permits up to four unpaired bases within the mature miRNA region) were applied to ensure the biological plausibility of the predicted secondary structures.

### 2.3 RNA-seq workflow for transcriptome assembly and novel transcript identification

To isolate mRNAs and ncRNAs, the rRNAs were eliminated. The mRNAs and ncRNAs were then fragmented into smaller pieces using fragmentation buffer and subsequently reverse transcribed into cDNA with random primers. Second strand cDNA synthesis was carried out using DNA polymerase I, RNase H, dNTPs (substituting dUTP for dTTP), along with the appropriate buffer. The stranded RNA-seq libraries were prepared using the VAHTS Universal V6 RNA-seq Library Prep Kit for Illumina (Vazyme, #NR604), following a protocol that yields RF-oriented reads. The resulting cDNA fragments were subsequently purified using the QiaQuick PCR extraction kit (Qiagen, Venlo, The Netherlands), followed by end repaired, poly(A) tail addition, and ligation of Illumina sequencing adapters. To finalize the process, UNG (Uracil-N-Glycosylase) was employed to remove the second strand cDNA. The digested products were subjected to size selection through agarose gel electrophoresis, followed by PCR amplification. Sequencing was performed on the Illumina HiSeqTM 4000 platform with services provided by Gene Denovo Biotechnology Co. (Guangzhou, China).

Raw reads encompassing adapter sequences or low quality bases were excluded, as they could interfere with subsequent analyses. To ensure high quality clean reads, sequences holding adapter sequences were first removed. Additionally, reads with over 10% of unknown nucleotides (N) or those with more than 50% of low quality bases (Q-value ≤ 20) were filtered out. To map reads to the ribosomal RNA (rRNA) database, the short reads alignment tool Bowtie2 (version 2.2.8) was utilized, as described by Langmead and Salzberg (2012). Reads aligned to rRNA were discarded, and the remaining sequences were used for transcriptome assembly and ensuing analysis. An index of the reference genome was generated, and paired end clean reads were aligned to the oil palm reference genome using HISAT2 (version 2.1.0) with the “-rna-strandness RF” option, while other parameters were kept at their default settings (Daehwan et al., 2015). Transcript reconstruction was performed using Stringtie (version1.3.4) software (Pertea et al., 2015), which, in combination with HISAT2, enables the identification of novel genes and splice variants of existing genes. To detect novel transcripts, all reconstructed transcripts were mapped to reference genome and were assigned to twelve groups with the help of Cuffcompare (Trapnell et al., 2010).Transcripts with one of the classcodes “u, i, j, x, c, e, o” were defined as novel transcripts. We used the following parameters to identify reliable novel genes: the length of transcript was longer than 200 bp and the exon number was more than 1. The Cuffcompare classification codes retained for analysis, u, i, j, x, c, e, and o, were selected based on their biological relevance in identifying potential novel transcripts that diverge from known annotations. These codes correspond to the following transcript categories: u: Intergenic transcripts, i: Intronic transcripts, j: Potentially novel isoforms, x: Exonic overlap with reference on the opposite strand, c: Transcripts contained within a reference transcript, e: Single-exon antisense transcripts, o: Generic exonic overlap with a reference transcript. These transcripts were subsequently aligned to the Nr, KEGG, and GO database for protein annotation.

To assess the protein coding potential of novel transcripts, we employed three software tools: CNCI (version 2), CPC (version 0.9-r2) (http://cpc.cbi.pku.edu.cn/), and FEELNC (version v0.2) (https://github.com/tderrien/FEELnc), using default settings. LncRNAs were identified by selecting transcripts that showed nonprotein coding potential in both CNCI and CPC analyses. The identification of lncRNAs was based on the consensus of three software tools (CNCI, CPC2, and FEELnc), all of which had to predict a transcript as non-coding. Additionally, transcripts were required to have a length ≥ 200 bp and at least one exon. These lncRNAs were then categorized into five types based on their positional relationship to protein coding genes: intergenic, bidirectional, intronic, antisense, and sense overlapping lncRNAs. Each type of lncRNAs may perform a distinct biological role.

### 2.4 Identificationof DEGs, DELs, and DEMs

StringTie v1.3.1 was used to assemble the mapped mRNAs and lncRNAs. The expression levels and variations of each transcription were quantified by calculating the FPKM (fragment per kilobase of transcript per million mapped reads) for each transcriptional region. The variation in magnitude of mRNA and lncRNA expression between cold stress treated and CK oil palm was determined by dividing the FPKM value of cold stress treated oil palm by the FPKM value of the CK oil palm. miRNAs expression levels were determined and normalized using transcripts per million (TPM). The TPM value was calculated as follows.


$$\text{TPM}=\text{actual miRNA counts}/\text{total counts of clean tags}\times 10^{6}$$

The fold change in miRNA expression between CK and cold stress treatment oil palm was calculated using:


TPM of cold stress treatment oil palm/TPM of CK oil palm

Raw count matrices were used for DESeq2 (mRNA/lncRNA) with size-factor normalization; small-RNA counts analyzed with edgeR (TMM). FPKM/TPM were used only for expression visualization (Love et al., 2014). DEGs and DELs were screened using the false discovery rate (FDR) for multiple testing correction. In contrast, DEMs were identified based on uncorrected p-values. This approach was adopted due to the characteristically low abundance of miRNAs, as using p-values in the initial screening helps reduce the risk of missing miRNAs with potential biological importance.

### 2.5 Predicting lncRNA target genes using computational tools

Candidate target genes of DEMs were identified using PatMatch software (Version 1.2) (Yan et al., 2005). The prediction of *cis*- and trans-target genes for DELs followed the methodology outlined by Wu et al. (2019) (Wu et al., 2019). To identify cis-target genes of DELs, coding genes located within a 10 kb region upstream and downstream of the DELs were analyzed. The strength and direction of the relationship between the expression levels of DELs and genes can be assessed using Pearson correlation coefficients. Genes showing a correlation coefficient with an absolute value exceeding 0.95 are considered strongly correlated and are predicted to be trans-target genes of DELs. Correlation analysis was conducted using all 18 samples collected across the time points. Although the resulting p-values were not adjusted for false discovery rate (FDR), the selected trans-regulatory pairs exhibited highly significant p-values (**Supplementary Table S1**). Additionally, certain antisense lncRNAs can modulate the expression of their corresponding sense transcripts by forming sense-antisense pairs, as described by Zhao et al. (2018) (Zhao et al., 2018). RNAplex software was employed to predict the complementary interaction between differentially expressed antisense lncRNAs and genes, helping to identify potential interactions (Tafer and Hofacker, 2008). The parameters for the RNAplex software were set as -e -30.

### 2.6 Exploring the role of DELs in miRNA-mediated gene regulation networks

To explore DELs as potential precursors of DEMs, we performed a Blast search by aligning the DEL sequences to the miRbase database (Release 22) (Griffiths-Jones et al., 2006). DELs exhibiting over 90% sequence similarity were considered potential miRNA precursors. Additionally, the miRPara software was employed to further predict DEM precursors from the identified DELs (Wu et al., 2011).

To identify interactions between differentially expressed DELs and DEMs, we performed target prediction using psRNATarget (2017 release) with an expectation cutoff of 2.5 (Dai et al., 2018). The default scoring scheme was applied, which imposes stronger penalties for mismatches within the seed region (positions 2–8). Although no additional filters were used during initial prediction beyond the three endogenous target mimic (eTM) rules, candidate pairs were further prioritized based on a significant negative correlation (Pearson “r” < –0.7, “p” < 0.05) between the miRNA and its predicted target. To predict DELs acting as eTMs for DEMs, TAPIR was employed under criteria proposed by Wu et al. (2013), which require: (i) a three-nucleotide bulge permitted only between positions 9–12 from the 5′ end of the miRNA; (ii) perfect complementarity between positions 2–8; and (iii) no more than three total mismatches and/or G/U pairs outside the bulge region. Based on these predictions, regulatory networks integrating DEGs, DELs, and DEMs were constructed. mRNA–lncRNA pairs were identified through antisense, cis-, or trans-regulation; mRNA–miRNA pairs were selected based on targeting relationships supported by Pearson correlation (“r” < –0.7, “p” < 0.05); and miRNA–lncRNA pairs were included if they showed targeting relationships (“r” < –0.7, “p” < 0.05) or were predicted as precursor-derived interactions. All subnetworks (DEL–DEG, DEM–DEG, DEM–DEL) were visualized using Cytoscape (v3.8.0), and an integrated DEL–DEM–DEG regulatory network was subsequently assembled.

### 2.7 Analysis of function and pathway enrichment

GO and KEGG pathway enrichment analyses of DEGs, as well as the targets of DELs and DEMs, were performed with the aid of OmicShare online platform (http://www.omicshare.com/tools). The p-value obtained were adjusted using FDR correction, with a threshold of FDR ≤ 0.05 considered indicative of significant enrichment. The statistical method used was the hypergeometric test, with the complete set of genome genes serving as the background gene set. For the raw p-values obtained from the hypergeometric test, we applied the Benjamini-Hochberg (BH) method for false discovery rate (FDR) correction. All p-values reported in the study are FDR-adjusted p-values, with the significance threshold set at FDR < 0.05. The species database version was GCF_000442705.1 (https://www.ncbi.nlm.nih.gov/datasets/genome/GCF_000442705.1/).

### 2.8 PCR profiling of gene expression under cold stress

cDNA was synthesized using 1μl RNA with MightyScript first-strand cDNA synthesis kit (gDNA digester) (Sangon Biotech, Shanghai, China), by following manufacturer’s instructions. Quantitative real-time PCR was performed following the standard protocol provided with the SYBR Premix Ex Taq™ kit (TaKaRa), using 384-well optical plates (Axygen) and a final reaction volume of 10 μl. The real-time qPCR reactions were carried out in 0.2 ml tubes using a Mastercycler ep realplex4 (Eppendorf) instrument with the following thermal cycling conditions: 30 seconds at 95°C, 15 seconds at 55°C and 20 seconds at 65°C. The reaction terminated with a melt curve analysis, gradually increasing the temperature from 60 to 95°C over 20 minutes to confirm the specificity of the PCR. All qPCRs reactions were performed in both biological and technical triplicates. The final Ct values were calculated as the average of nine measurements. Primer amplification efficiencies were between 93% and 98%. The 2^-ΔΔCt^ method was adopted for analyzing the expression levels and the expression levels of genes were normalized to those of *ELF*, which was previously found to be a stable reference gene under abiotic stress (Xia et al., 2014). One-way ANOVA with Tukey’s HSD *post hoc* test (p < 0.05) was conducted to assess the significant difference in expression levels across different time points following cold treatment. SPSS software was used to analyze the expression data. Primers for this experiment are provided in **Supplementary Table S2**.

### 2.9 Analysis of the *cis*-acting element of the miR156-z promoter

Using the 2 kb region upstream of the transcription start site of miR156-z as the default promoter region (NC_025994.1:18728248-18730247(+)), the PlantCARE website (http://bioinformatics.psb.ugent.be/webtools/plantcare/html/, accessed on 4 March 2025) was utilized to analyze the *cis*-acting regulatory elements within the miR156-z promoter.

### 2.10 Amplification of miR156-z precursor sequence

To ensure the proper folding of the miRNA precursor, approximately 200 bp segments were added to both the 3’ and 5’ ends of the miR156-z precursor sequence. Specifically, Xba I and Kpn I restriction enzyme sites were incorporated at the 5’ end of the primers. The amplification primers were as follows: miR156-z-F: 5’-GCTCTAGAGCGGATCTTGGTCAAGTGTG-3’, miR156-z-R: 5’-ACACAACACCG AAGCTTGGTGATGTGAC–3’. Total DNA was extracted from oil palm leaves, and PCR was conducted with a total reaction volume of 20 μL in 0.2 mL tubes under the following conditions: 95°C for 2 min, 94°C for 30 s, 58°C for 30 s, 72°C for 30 s, and 72°C for 5 min. The gel extraction and purification were performed using the TaKaRa Gel Recovery Kit, and the concentration of the extracted DNA was measured.

### 2.11 Plasmid preparation and recombinant vector construction

The miR156-z was ligated into the pEASY-T1 vector (T7/SP6) using the pEASY-T1 Cloning Kit, with the following reaction setup: 4 μL of the miR156-z precursor sequence and 1 μL of the pEASY-T1 Cloning Vector. The bacterial culture identified as correct by PCR was subjected to sequencing (**Supplementary Figure S1**). After comparing the sequencing results, the plasmid DNA was digested using Xba I (Takara, Cat#1093A) and Kpn I (Takara, Cat#1068A). The double digestion reaction was set up as follows: 1 μL of Xba I, 1 μL of Kpn I, 1 μg of DNA, 2 μL of 10X Mix, and ddH_2_O was added to bring the total reaction volume to 20 μL. The recovered product, the pxZP008 vector, and the miR156-z precursor fragment were then ligated to obtain the miR156-z overexpression vector. This vector was transformed into *E. coli DH5α* for the selection of recombinant plasmids. The selection concentration of kanamycin is 50 μg/mL. Finally, the plasmid was extracted from the bacterial culture.

### 2.12 Oil palm protoplast transformation

The culture of oil palm embryoids took place at 28°C, following a 12-hour light and 12-hour dark photoperiod, with sub-culturing occurring every 30 days. The yield of protoplasts isolated from per gram of oil palm embryogenic callus fresh weight was (5.8 ± 1.2) × 10^6^, with a viability of 79% ± 3% as determined by FDA staining. Approximately 2-3 μg of high-quality genomic DNA could be extracted from 200 μL of protoplasts at a concentration of 1 × 10^6^ mL^−^¹. The PEG solution used was 40% (w/v) PEG4000, composed of 40% PEG 4000, 0.2 M mannitol, and 0.1 M CaCl_2_. During transformation, the protoplasts were gently mixed with plasmid DNA and an equal volume of PEG solution, incubated at room temperature for 30 minutes, and then slowly diluted with W5 solution to terminate the reaction. The transformation efficiency was measured using qRT-PCR. The recombinant vector was transformed into oil palm protoplasts using PEG-mediated transformation, with the CK group being transformed with an empty vector and the treatment group being transformed with the YFP-zmmiR156-z fusion plasmid. All experiments were independently repeated three times, and the data are presented as mean ± SD. Statistical significance was analyzed using one-way ANOVA, and significant differences between groups are indicated with asterisks in the figures (*p < 0.05, **p < 0.01).

## 3 Results

### 3.1 High throughput identification of lncRNAs in oil palm exposed to cold stress

**Figure 1:**
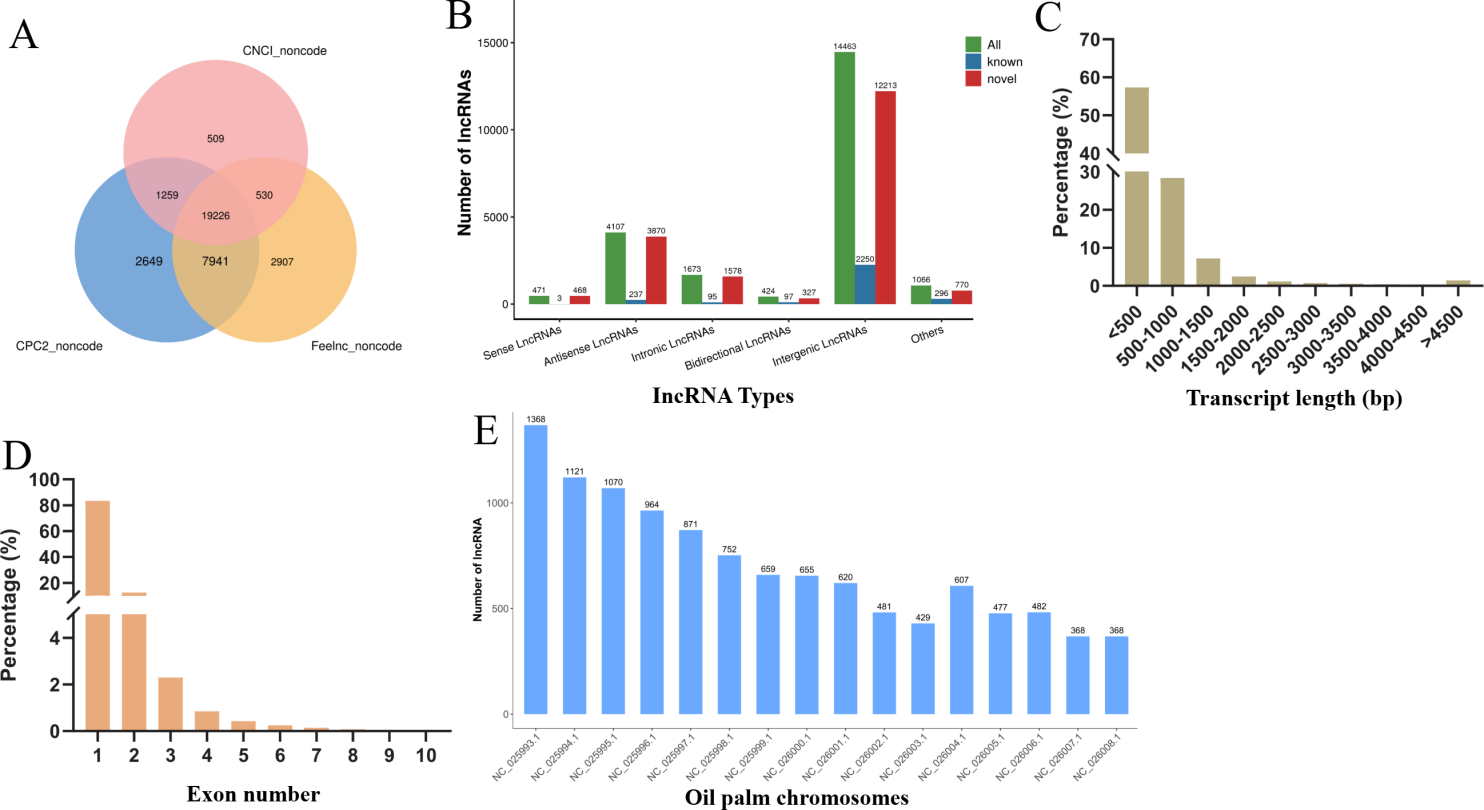
Identification and characterization of lncRNAs in oil palm. **(A)** Numbers of lncRNAs obtained by CNCI, CPC2 and Feelnc. **(B)** Types of the obtained lncRNAs. **(C)** Length distribution of the obtained lncRNAs. **(D)** Exon number distribution of the identified lncRNAs. **(E)** lncRNA distribution on oil palm chromosomes.

To explore lncRNAs in oil palm under low temperature conditions, we constructed eighteen cDNA libraries from leaf tissues (**Supplementary Table S3**). These included CK groups and groups subjected to varying durations of low-temperature stress (Cold 0.5, Cold 1, Cold 2, Cold 4, and Cold 8). High-throughput RNA sequencing generated an average of 12.37 Gb clean data from the CK library following quality control. The Cold treatment libraries produced 12.99 Gb, 14.90 Gb, 12.34 Gb, 13.18 Gb, and 13.51 Gb from the Cold 0.5, Cold 1, Cold 2, Cold 4, and Cold 8 libraries. The quality scores, Q20 and Q30 were consistently above 97.16% and 92.20%, with a mean GC content of 44.09% (**Supplementary Table S3**). After excluding transcripts shorter than 200 bp, we utilized CNCI, CPC2, and Feelnc to identify a total of 19,226 high-confidence lncRNAs (**Figure 1A**). Based on their genomic positions, these lncRNAs were sorted into six categories i.e., 471 sense, 4,107 antisense, 1,673 intronic, 424 bidirectional, 14,463 intergenic and 1,066 classified as ‘other’ (**Figure 1B**). The identified lncRNAs ranged in length from 302 to 18,058 bp with the median length of 566 bp, the majority (85.76%) being less than 1,000 bp and a smaller subset (4.61%) exceeding 2,000 bp (**Figure 1C**). The Regarding exon composition, most lncRNAs (83.41%) were single-exon, while 12.48% contained two exons, 2.30% contained three exons, and a minimal fraction (1.82%) possessed over three exons (**Figure 1D**). Through the analysis of lncRNAs distributed on oil palm chromosomes, it was found that chromosome NC_025993.1 contains the highest number, with 1368 lncRNAs, followed by chromosome NC_025994.1 (1121). Chromosome NC_026004.1 has the smallest number of lncRNAs, only 429 (**Figure 1E**).

### 3.2 miRNA profiling in oil palm under cold stress

**Figure 2:**
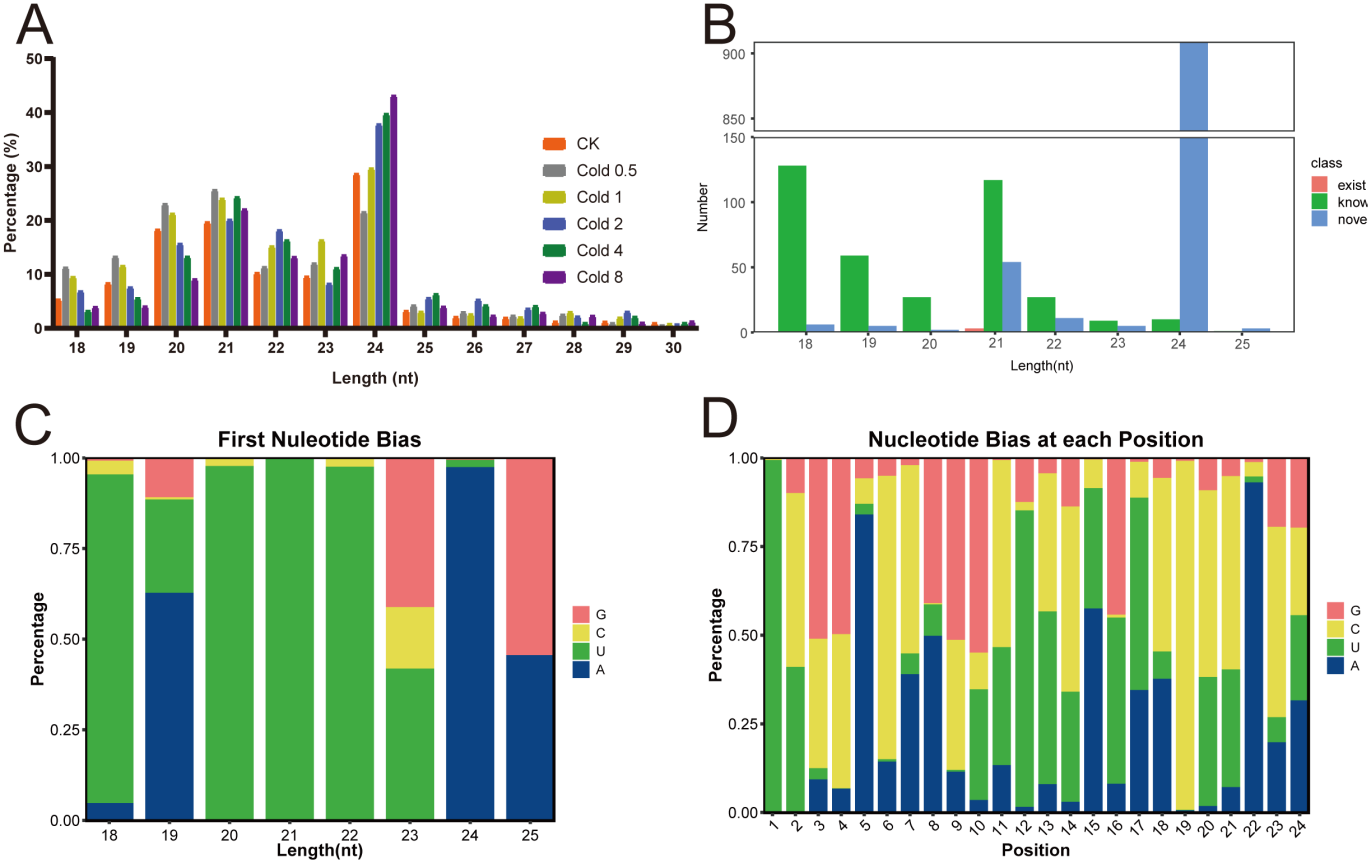
Identification and characterization of miRNAs of oil palm. **(A)** Length distribution of small RNA reads. **(B)** Length distribution of the obtained miRNAs. **(C)** Percents of nucleotide bias of miRNAs with different lengths. **(D)** Position distribution of different nucleotide bias.

To develop miRNAs for oil palm, we constructed eighteen sRNA libraries from the leaf tissues of both CK and plants subjected to cold stress, followed by sequencing. After rigorous quality control, including the removal of low-quality reads, poly A sequences, adapter sequences, and any reads shorter than 18 nt. As a result, the clean reads obtained from the CK, Cold 0.5, Cold 1, Cold 2, Cold 4, and Cold 8 libraries averaged 14.30, 11.63, 9.698, 15.03, 13.74, and 13.19 million, respectively (**Supplementary Table S4**). Analysis of the length profile of clean reads revealed that the major portion of sRNAs (83.61%) fell within the 20–24 nt range. We found that 24 nt sRNAs predominated, followed by 21 and 20 nt sRNAs (**Figure 2A**). The clean reads were further marked and classified into different categories, including rRNA, snRNA, snoRNA, tRNA, exon-sense, miRNA, and others. Comparative analysis with the CK group showed a higher proportion of known-miRNAs under the Cold 2 and Cold 4 treatments (**Supplementary Table S5**).

We obtained 2,376 miRNAs in total, comprising 1,382 known and 994 novel miRNAs. The miRNAs lengths spanned from 18 to 25 nt, among the known miRNAs, 33.86% were 18 nt long, while 52.47% of the novel miRNAs had a length of 24 nt (**Figure 2B**). Under cold stress, oil palm plants exhibited a significantly greater percentages of 21 and 22 nt miRNAs compared to the CK, accounting for 97.36% of the total miRNAs, while the percentage of 23 and 24 nt miRNAs remained almost identical between the CK and cold stress groups (**Supplementary Figure S2**). Analysis of the first nucleotide of miRNAs revealed a strong enrichment for uracil (U), accounting for 99.11% (**Figure 2C**, **Supplementary Table S6**). Specially, the first position in miRNAs (18, 20, 21, and 22 nt of lengths) was most commonly occupied by U, with percentages of 90.66%, 97.48%, 99.70%, and 97.61%, respectively. Additionally, nucleotide bias analysis across each position within the miRNAs revealed that cytosine (C) had the highest percentage at 34.97%, followed by U at 28.57%, adenine (A) at 19.01%, and guanine (G) at 17.45% (**Figure 2D**).

### 3.3 Cold stress induced gene expression and regulatory pathways in oil palm

**Figure 3:**
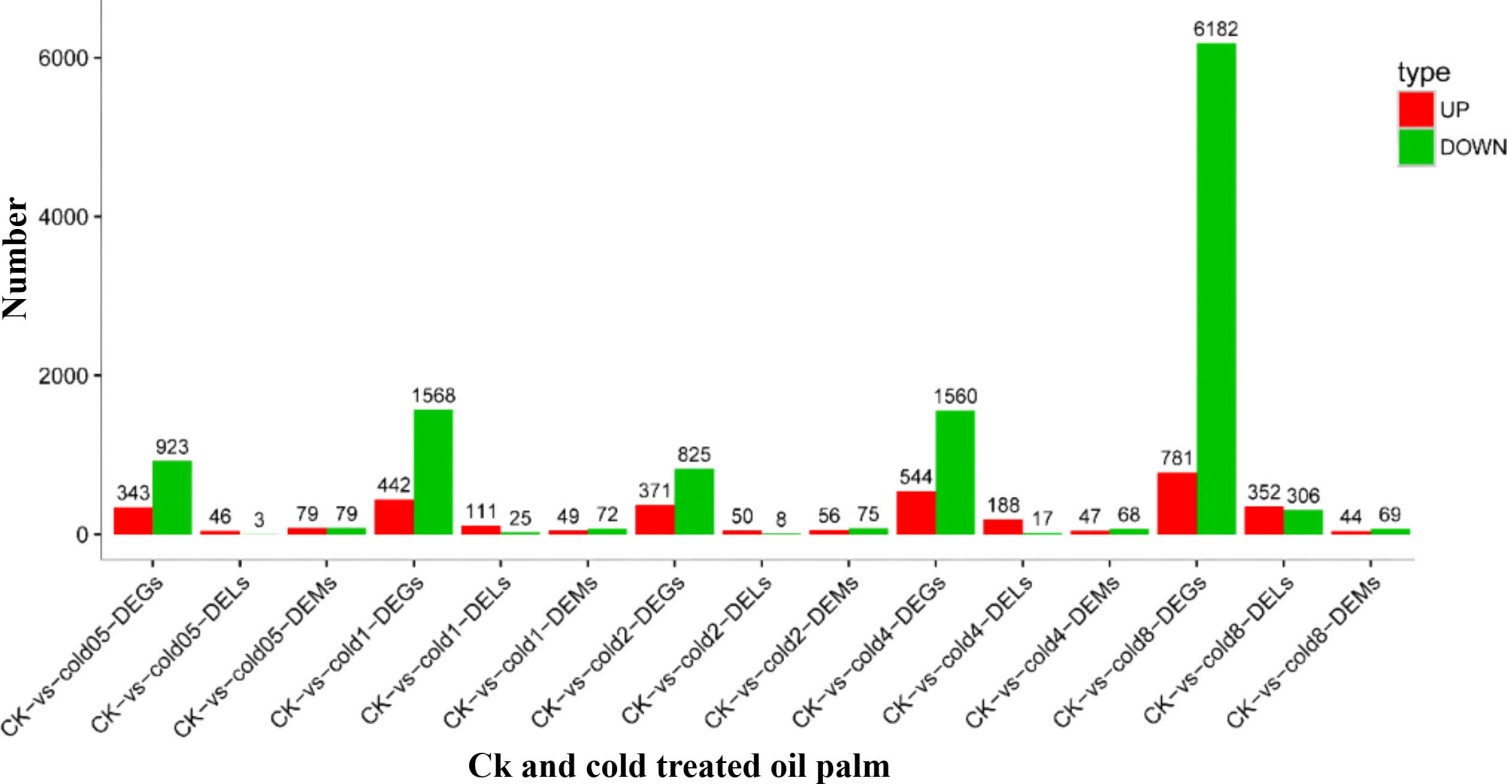
Number of DEGs, DEMs and DELs identified in CK and cold treated oil palm.

Differential expression analysis identified 13,539 DEGs, with 2,481 up-regulated and 11,058 down-regulated ones (**Figure 3**, **Supplementary Table S7**). Subsequent GO enrichment analysis revealed significant enrichment of these DEGs across 40 GO terms (p < 0.05): 23 biological process terms, primarily related to cellular and metabolic processes; 14 molecular function terms, largely linked with binding and catalytic actions; and 3 cellular component terms, focusing on cellular features and protein-based complexes (**Supplementary Figure S3**).

KEGG enrichment profiling identified associations between the DEGs and 121 distinct pathways. Prominently, 11 of these pathways were highly prevalent, primarily within the domain of metabolism. Additionally, 4 pathways were associated with the processing of genetic information, two were related to the processing of environmental information, and one was connected to cellular functions and organismal systems. The three most significantly enriched pathways (p < 0.05) were identified as carbohydrate metabolism, lipid metabolism, and the metabolism of vitamins and cofactors (**Supplementary Figure S4**, **Supplementary Table S8**). Within the top enriched pathways related to carbohydrate metabolism, lipid metabolism, and the metabolism of vitamins and cofactors, we have included key genes such as *TPI1* (*LOC105044400*), *GAPDH* (*LOC105059882*), *SAD* (*LOC105049737*), *FADs* (*LOC105054111*), *RDH5* (*LOC105044453*), and *RBP4* (*LOC105049664*) being highly expressed.

The comparative analysis between the CK and cold stress treatment groups in oil palm yielded a total of 638 differentially expressed miRNAs (DEMs), with 275 activated and 363 repressed ones (**Figure 3**, **Supplementary Table S9**). To elucidate the biological processes and molecular functions mediated by these DEMs, we inferred their potential target genes that led to the identification of 2,565 genes targeted by 35 distinct DEMs (**Supplementary Table S10**).

GO enrichment analysis classified these target genes into 22 GO terms including 13 biological functions, 3 cellular elements, and 6 molecular processes (**Supplementary Figure S5**). Among the biological functions, ‘cellular activity’ was the largest group, encompassing 29 genes, followed by ‘metabolic process’ with 26 genes, and ‘biological regulation’ with 11 genes. In the cellular elements category, ‘cellular organization’ was the leading GO term, representing 11 genes. In the molecular processes class, the two most prominent GO terms were ‘binding’, which included 33 genes, and ‘catalytic activity’, with 26 genes. Additionally, differentially expressed target genes were subjected to KEGG enrichment analysis to further understand their biological functions. The predicted targets genes were associated with 23 distinct KEGG pathways, with a significant enrichment observed in ‘signal transduction’, ‘biosynthesis of secondary metabolites’ and ‘metabolic pathways’ (**Supplementary Table S11**). Within the top enriched pathways related to signal transduction, biosynthesis of secondary metabolites and metabolic pathways, we have included key genes such as *PYL* (*LOC105048209*), *PP2C* (*LOC105045917*), *MYB* (*LOC105059999*), *bZIP* (LOC105060907), *bHLH* (*LOC140856477*), and *AP2/ERF* (*LOC105041281*) being highly expressed. The study identified a total of 1,106 DELs in oil palm plants subjected to cold stress compared to CK plants. These DELs included 747 activated and 359 repressed lncRNAs (**Figure 3**, **Supplementary Table S11**). To investigate the roles of these DELs, we inferred their potential antisense, cis-acting and trans-acting target genes. From the analysis, 432 potential target genes were identified as being regulated by 168 DELs, which included 61 antisense target genes (**Supplementary Table S12**), 300 *cis*-target genes (**Supplementary Table S13**), and 90 trans-target genes (**Supplementary Table S14**). According to the GO enrichment analysis, the potential targets of DELs were assigned to 25 distinct GO terms, encompassing 14 biological functions, 3 cellular elements and 8 molecular processes (**Supplementary Figure S6**). Specifically, the metabolic process and cellular activities were the most abundant categories within the biological functions. In the cellular elements category, the term ‘cellular organization’ was the most prevalent, while in the molecular processes category, ‘catalytic activity’ and ‘binding’ were the top two GO terms. Furthermore, KEGG pathway enrichment analysis showed that the likely candidates of DELs were associated with 30 distinct pathways. These targets were especially prominent in pathways associated with metabolic activities, the synthesis of secondary metabolites, carbon metabolism, ribosome, interactions between plants and pathogens, and plant hormone signaling pathways (**Supplementary Table S15**).

### 3.4 DEL-DEM-DEG regulatory networks in cold stress response and gene interactions

**Figure 4:**
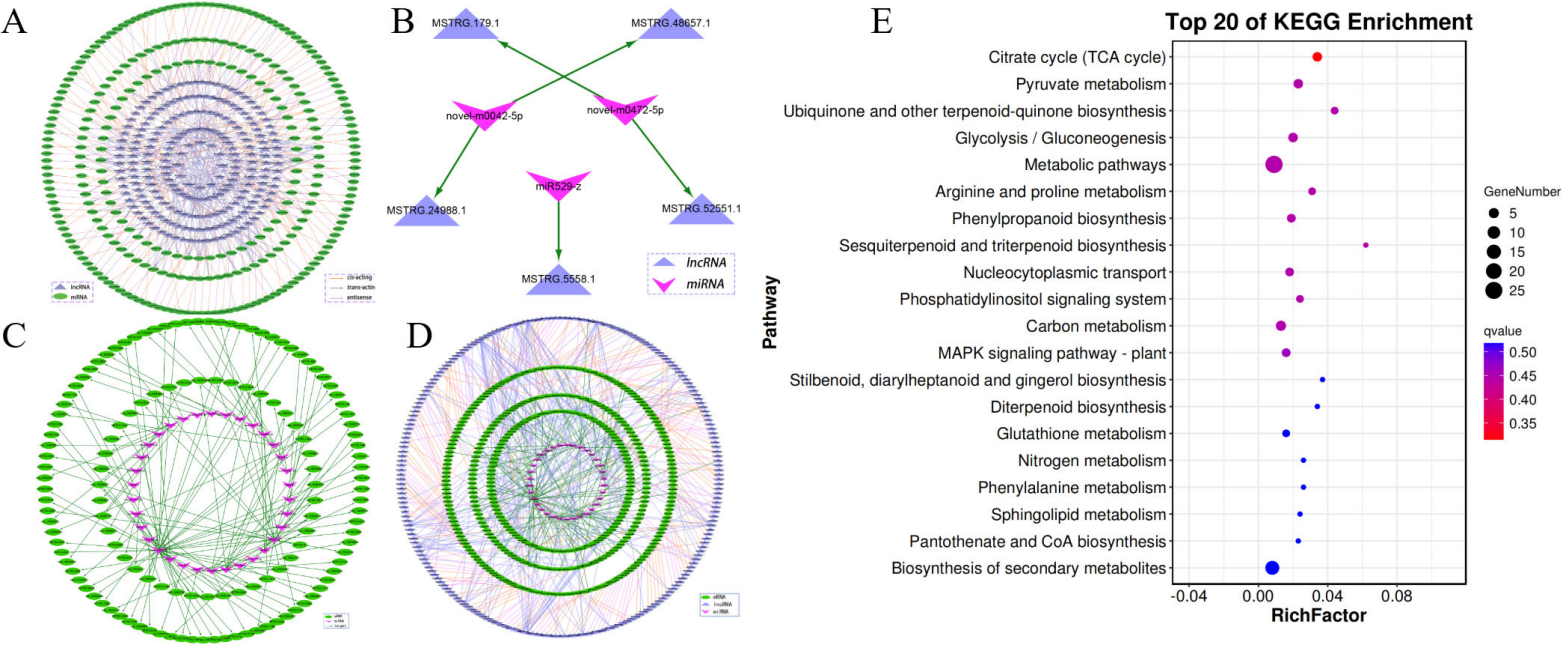
DEL-DEM-DEG regulatory networks involved in cold stress. **(A)** Network of DEL-DEG related to cold treated oil palm. **(B)** The DEL-DEM regulatory network related to cold treated oil palm. **(C)** The DEM-DEG network involved in cold treated oil palm. **(D)** The DEG-DEM-DEL regulatory network involved in cold treated oil palm. **(E)** The top 20 enriched KEGG pathways of DEGs in the DEG-DEM-DEL regulatory network.

Of the DEL targets, 272 genes were found to coincide with DEGs. These DEGs, alongside their associated 44 DELs were selected to build a regulatory network between DEL and DEG. The network consisted of 316 nodes and 203 connections, which included 35 antisense, 102 *cis*-regulatory, and 66 trans-regulatory interactions (**Figure 4A**). LncRNA are known for their role in modulating target gene expression and can function as precursors, targets, or enhancers of miRNAs (eTM) (**Supplementary Table S16**). To gain a deeper understanding of how lncRNAs influence regulatory networks in response to cold stress, we predicted the interactions between DELs and DEMs. As a whole, 3 DELs were found to serve as precursors for 3 DEMs, 23 DELs were identified as targets of 8 DEMs, and 17 DELs acted as eTMs of 4 DEMs. Using these interactions, a DEM-DEL regulatory network was developed. **Figure 4B** illustrates that the network included 8 nodes, comprising 3 DEMs and 5 DELs. Besides the interactions between lncRNA and miRNA, miRNAs also performed a task in regulating target genes expression. The DEM-DEG network included 34 DEMs and 134 DEGs (**Figure 4C**).

To explore the interactions between mRNAs, miRNAs and lncRNAs under cold stress, a DEG-DEM-DEL regulatory network was developed. As shown in **Figure 4D**, this network comprised 402 DEGs, 214 DELs, and 34 DEMs. Within the network, the hub nodes (genes, lncRNAs, and miRNAs) with a degree higher than 10 were identified and are considered key regulators in the cold stress response. For instance, MSTRG.10236.1 exhibited 23 connections, followed by MSTRG.10711.1 (degree = 21), MSTRG.12095.1 (degree = 18), MSTRG.1327.1 (degree = 17), and MSTRG.1504.1 (degree =14). Additionally, miR156-z (degree = 13) and miR395-y (degree = 11), along with the ncbi_105033424 (degree = 16), were also recognized as hubs. These key regulators likely perform substantial tasks in the adaptation to cold stress and may represent promising candidates for further functional analysis.

To examine the possible roles of the DEL-DEM-DEG regulatory networks, GO and KEGG pathway enrichment analyses were performed on the DEGs within the network. The DEGs were categorized into 28 GO terms, embracing 16 biological functions, 9 molecular processes, and 3 cellular elements (**Supplementary Figure S7**). The most prevalent GO term was ‘abiotic stress response’, which suggests that these genes (e.g., FADs, Fatty Acid Desaturase Genes; PAL, lipid pathway genes; LOXs, Lipoxygenase Genes; PLD, Phospholipase Genes) might be essential for the cold stress adaptation process. The KEGG pathway analysis identified 43 pathways with significant enrichment of DEGs, highlighting ‘metabolic pathways’ (ko01100), ‘biosynthesis of secondary metabolites’ (ko01110), and ‘carbon metabolism’ (ko01200), as the most prominently enriched categories. These results imply that the DEL-DEM-DEG regulatory networks are likely to be integral to metabolic and biosynthetic processes that facilitate the response to cold stress (**Figure 4E**, **Supplementary Table S17**).

### 3.5 DEL-DEM-DEG network analysis related to palm oil synthesis

**Figure 5:**
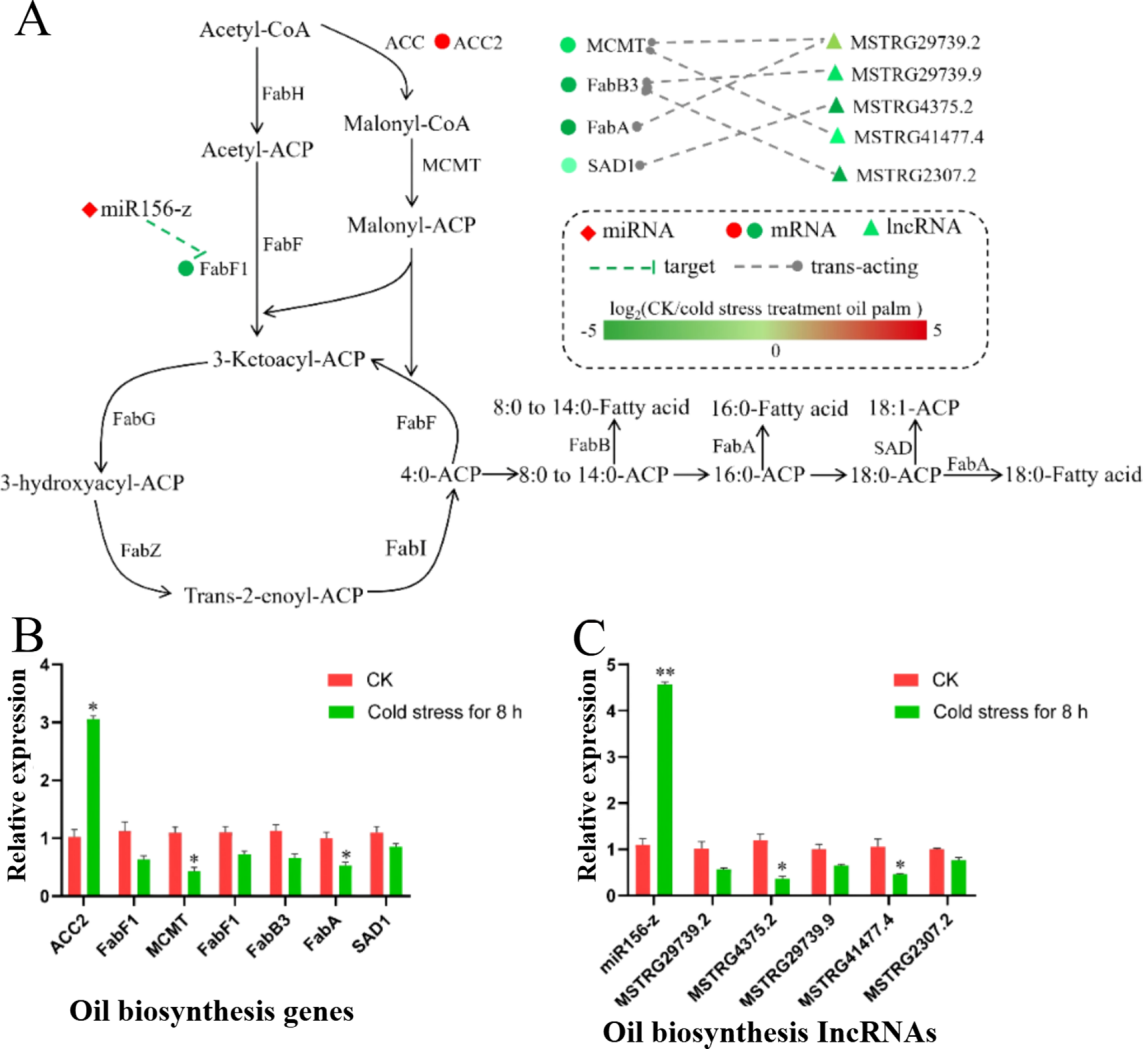
Expression profiles of DEGs, DEMs and DELs involved in palm oil biosynthesis. **(A)** Expression patterns of DEGs, DEMs and DELs related to palm oil biosynthesis from RNA-seq data. The color intensity of the shape represents the log2 fold change (cold treated/CK), as the color scale. **(B, C)** qRT-PCR validation of DEGs, DEMs and DELs involved in palm oil biosynthesis. One-way ANOVA was used to test significant difference (Tukey’s HSD, p < 0.05). Values were the means ± SEM of three replicates. ** for p < 0.01 and * for p < 0.05.

To investigate the effect of cold stress on palm oil synthesis, DELs, DEMs, and DEGs related to this process were identified and analyzed. Specifically, the expression of seven genes involved in the synthesis pathway was observed to change under cold stress conditions. These included *ACC2* (Acetyl-CoA carboxylase), *FabF1* (β-ketoacyl-ACP synthase II), *MCMT* (Malonyl-CoA: Acyl Carrier Protein Malonyltransferase), *FabB3* (β-ketoacyl-ACP synthase II), *FabA* (β-hydroxydecanoyl-acyl carrier protein dehydratase), and *SAD1* (Stearoyl-Acyl Carrier Protein Desaturase). Notably, based on the expression data, *ACC2* exhibited up-regulated, whereas the remaining genes were down-regulated. Researchers also identified miRNAs, lncRNAs, and mRNAs that were implicated in the regulation of palm oil synthesis under cold stress. For example, miR156-z was found to be up-regulated and appeared to negatively regulate the *FabF1* gene. Additionally, five predicted lncRNAs (*MSTRG*.*29739.2*, *MSTRG*.*29739*.9, *MSTRG*.*4375*.*2*, *MSTRG*.*41477.4*, and *MSTRG*.*2307.2*) were identified as negatively regulating genes involved in palm oil synthesis, including *MCMT*, *FabB3*, *FabA*, and *SAD1*. The specific targeting predictions were as follows: *MSTRG*.*29739.2* was predicted to target both *MCMT* and *FabA*, *MSTRG*.*29739.9* to target *FabB3*, *MSTRG.4375.2* to target *SAD1*, *MSTRG.41477.4* to target *MCMT*, and *MSTRG.2307.2* to target *FabB3* (**Figure 5A**). Furthermore, the expression profiles of the aforementioned DELs, DEMs, and DEGs were corroborated through qRT-PCR (**Figures 5B, C**). The findings, which aligned with the RNA-seq data, collectively indicate that cold stress may inhibit palm oil synthesis by modulating the expression of crucial genes involved in the synthesis pathway.

### 3.6 Analysis of the *cis*-acting elements of the miR156-z promoter

The 2 kb region upstream of the transcription start site of miR156-z was analyzed to identify *cis*-acting elements that may regulate its expression. The analysis revealed the presence of hormone-responsive elements (Abscisic acid (ABA) response elements (4), Methyl jasmonate (MeJA) response elements) (6) and stress-responsive elements (Light-responsive elements, Low-temperature-responsive elements) (12) (**Supplementary Table S18**), indicating that miR156-z expression is likely influenced by hormonal signals and environmental stresses. These findings provided a foundation for further studies on the regulatory mechanisms and biological functions of miR156-z in plants.

### 3.7 Construction of expression vector

**Figure 6:**
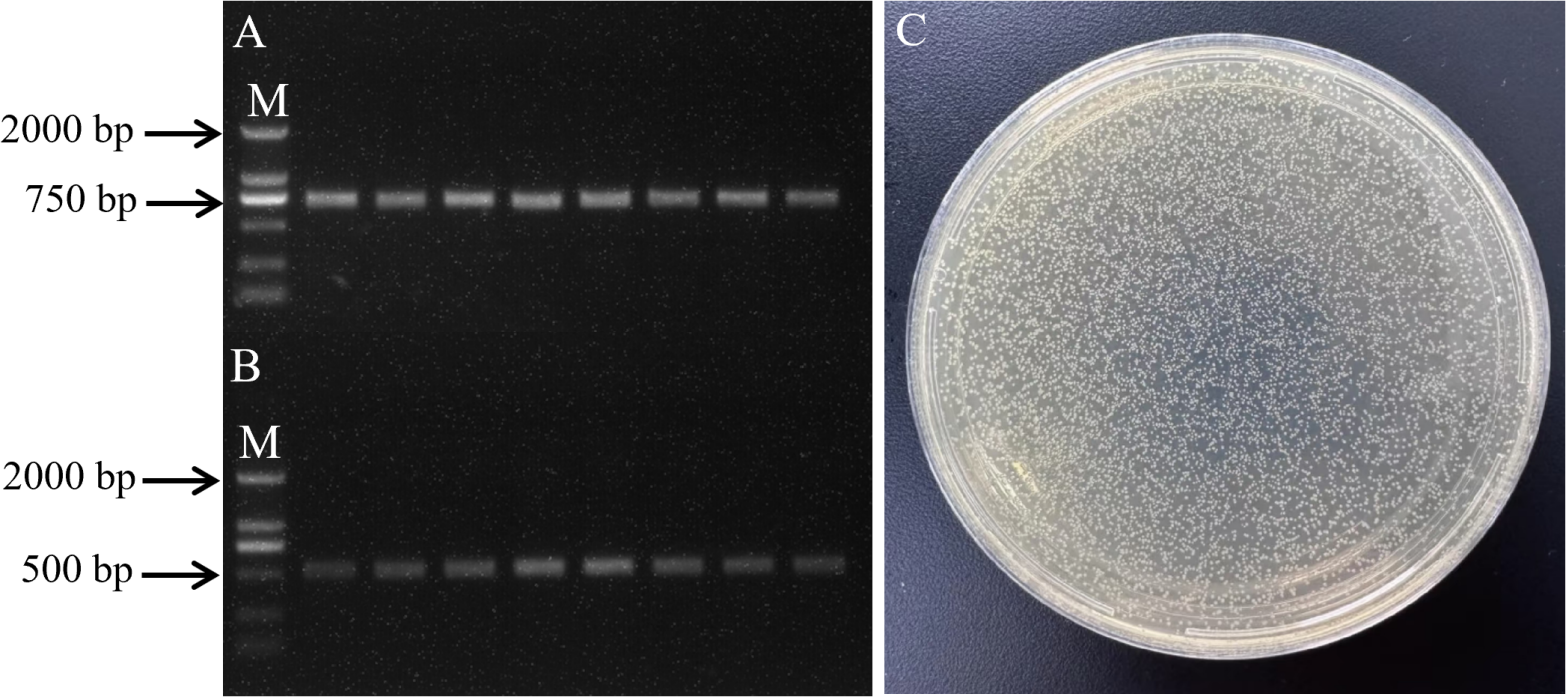
Transformation of *E. coli DH5α* and PCR amplication. **(A)** PCR amplication of oil palm miR156-z precursor sequences. **(B)** PCR identification of YFP-miR156-z recombinant vector bacterial broth. M means 2000 bp marker. **(C)** Transformation of *E. coli DH5α* with YFP-miR156-z recombinant vector.

Genomic DNA was extracted from oil palm leaves, and a fragment of approximately 510 bp was amplified by adding 200 bp upstream and downstream of the miR156-z precursor sequence. The size of the amplified band matched the expected size as per the experimental design. The amplified band was gel-purified and ligated into the pEASY-T1 Cloning Vector. Positive colonies were selected and cultured in LB medium containing ampicillin. PCR identification and sequencing of the bacterial culture confirmed that the amplified band matched the size of the miR156-z precursor sequence (**Figure 6A**). Plasmid DNA was extracted, and both the miR156-z plasmid and the pXZ008 vector were subjected to double digestion. The concentrations of the gel-purified digestion products were 63.2 ± 1.13 ng/μL and 14.8 ng/μL, respectively (**Supplementary Table S19**). The digested miR156-z and pXZ008 vector were ligated at a molar ratio of 1:3. The ligation product was then transformed into *E. coli DH5α* (**Figure 6B**). PCR analysis of the bacterial culture yielded a band of approximately 510 bp (**Figure 6C**), confirming the successful ligation of miR156-z into the pXZ008 vector. To functionally validate the constructed overexpression vector, its efficacy was tested in planta. qPCR analysis revealed that transient expression of the vector led to a significant overexpression of miR156-z (3 biological replicates; 25.8 ± 2.4-fold change relative to empty vector control; unpaired two-tailed t-test; p < 0.0001). Consequently, this overexpression resulted in significant repression of the miR156-z target gene *FabF1* (n = 6 biological replicates; 0.22 ± 0.03-fold change relative to control; unpaired two-tailed t-test; p = 0.0002). p-values were adjusted for multiple comparisons using the Holm-Šidák method.

The chromatin context represents a major limitation of our approach. Transient overexpression delivers the lncRNA transcript in trans, thereby completely decoupling it from its native genomic locus. For lncRNAs that function primarily in *cis*, such as those acting through transcription-dependent effects on local chromatin architecture or via allele-specific regulation, our assay system would not be expected to capture their biological activity, potentially resulting in false negatives. This constraint is a well-recognized aspect of such overexpression assays when applied to *cis*-regulatory elements. Furthermore, we acknowledge the important issue of tissue and cell type specificity. The function of many lncRNAs depends critically on cell-type-specific co-factors, including particular combinations of RNA-binding proteins or chromatin-modifying complexes. Therefore, the lack of an observed phenotype in our experimental system does not necessarily rule out a functional role for these lncRNA-gene pairs in their physiologically relevant tissue contexts. Furthermore, we acknowledge the important issue of tissue and cell type specificity. The function of many lncRNAs depends critically on cell-type-specific co-factors, including particular combinations of RNA-binding proteins or chromatin-modifying complexes. Therefore, the lack of an observed phenotype in our experimental system does not necessarily rule out a functional role for these lncRNA–gene pairs in their physiologically relevant tissue contexts.

### 3.8 Analysis of target gene *FabF1* expression in miR156-z overexpressing oil palm protoplasts

**Figure 7:**
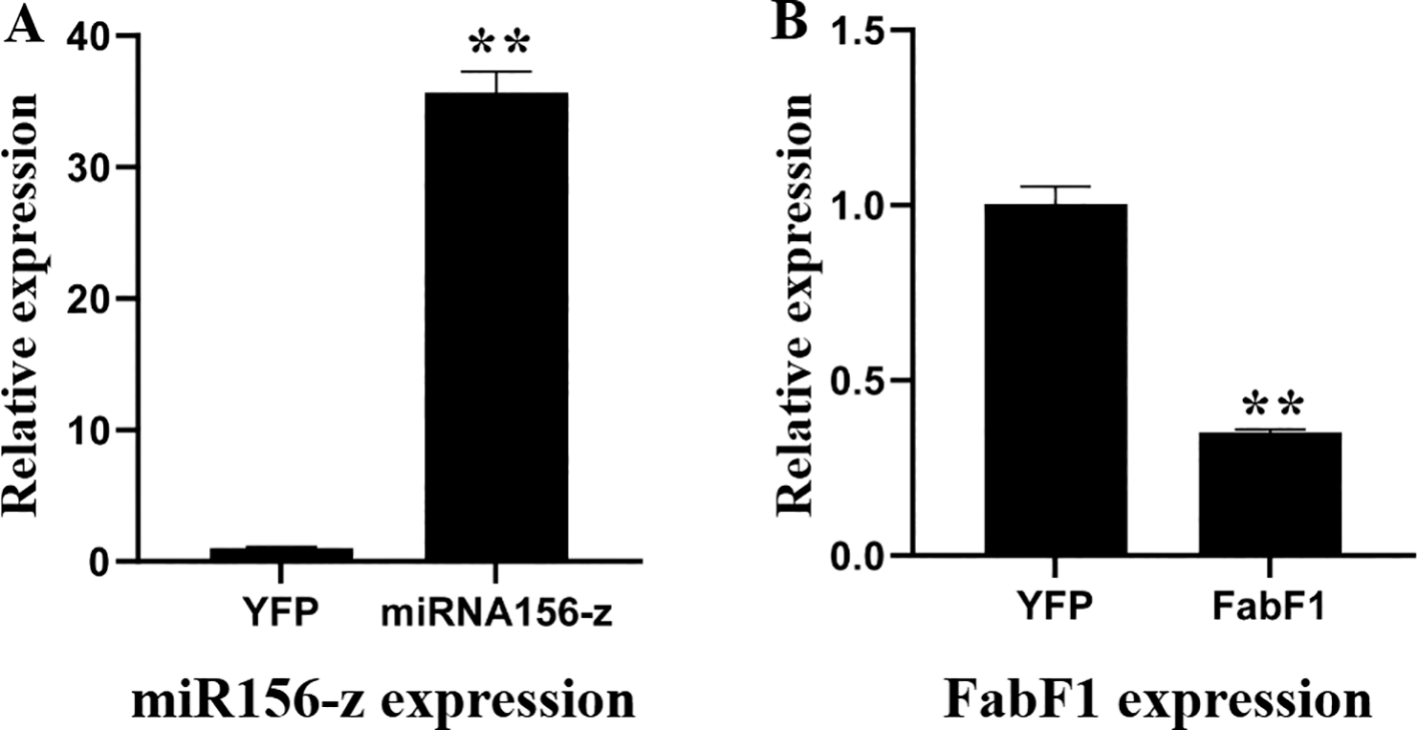
Expression analysis of miR156-z and FabF1 in CK and transgenic protoplast. **(A)** Detection of miR156-z expression by qRT-PCR. **(B)** Changes of the expression levels of *FabF1* after overexpression of miR156-z. Values were the means ± SEM of three replicates. ** for p < 0.01.

The pXZ008-miR156-z fusion plasmid and the CK plasmid without the miR156-z insert were introduced into oil palm protoplasts. The transformed protoplasts were cultured in the dark at 25°C for 3 days to allow for gene expression. Protoplasts transformed with the pXZ008-miR156-z fusion plasmid showed significantly higher expression of miR156-z compared to those transformed with the pXZ008 empty vector (n = 3 biological replicates; unpaired two-tailed t-test; p < 0.001) (**Figure 7A**). In order to investigate the relationship between miR156-z and its potential target gene *FabF1* in oil palm protoplasts, The relative expression levels of *FabF1* were quantified. The results showed that In protoplasts overexpressing miR156-z, the expression level of *FabF1* was significantly reduced compared to CK (transformed with the pXZ008 empty vector) (3 biological replicates; unpaired two-tailed t-test; p = 0.003; p-values adjusted using the Holm-Šidák method) (**Figure 7B**) (**Supplementary Table S20**). The down-regulation of *FabF1* in response to miR156-z over-expression suggested a regulatory relationship between miR156-z and *FabF1*.

## 4 Discussion

**Figure 8:**
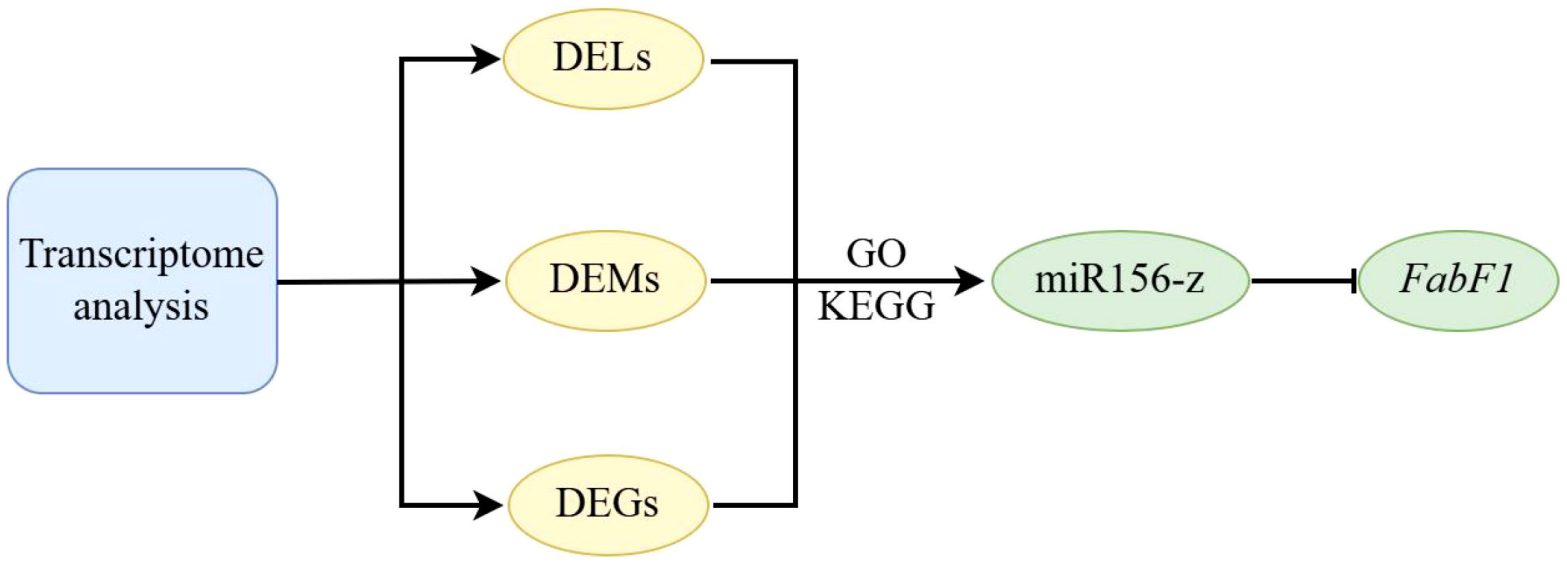
Schematic model depicting the contribution of differentially expressed genes (DEGs), miRNAs (DEMs), and lncRNAs (DELs) to enhanced cold tolerance in tropical oil palm.

Oil palm, as one of the world’s most productive oil crops, serves as a major source of edible oil widely used in food and processing industries across Asia, Africa, and Latin America (Gesteiro et al., 2019). Although miRNAs and lncRNAs play critical roles in post-transcriptional regulation and stress responses in plants (Sun et al., 2019; Paraskevopoulou and Hatzigeorgiou, 2016; Bridges et al., 2021), their functions in oil palm remain poorly understood, especially under cold stress (Somyong et al., 2016; Xia et al., 2020; Salgado et al., 2021, 2022; Saputra et al., 2023). To address this gap, this study constructed the first comprehensive ncRNA atlas of oil palm under cold stress, identifying key network hubs, including differentially expressed miRNAs, lncRNAs, and their target mRNAs, associated with lipid metabolism. A central lipid module was characterized, comprising critical genes such as *ACC2*, *FabF1*, *MCMT*, *FabB3*, *FabA*, and *SAD1*. Notably, the miR156-z→*FabF1* regulatory axis was validated as a key mechanism mediating cold inhibition of oil synthesis (**Figure 8**). These findings provide new insights into the ncRNA-regulated gene networks underlying cold stress response in oil palm, highlighting their role in modulating lipid biosynthesis.

To better understand the roles of miRNAs and lncRNAs in oil palm’s reaction to cold stress, we conducted a high-throughput RNA-seq analysis on leaves. This analysis was undertaken on oil palm leaves to thoroughly identify and depict miRNAs and lncRNAs pertaining to cold stress responses. In the course of this research, we identified 19,226 novel lncRNAs from oil palm subjected to cold stress conditions. A significant portion of these lncRNAs were found to be shorter than 1,000 bp and comprised of a single exon, aligning with observation made in other plant species (Liu et al., 2021). Among them, 1,106 lncRNAs exhibited differential expressed between CK and cold-stressed oil palm plants, indicating a potential involvement of lncRNA in the regulatory processes behind oil palm’s reaction to cold stress. This finding aligns with and expands upon earlier work by Xia et al. (2020), who used strand-specific RNA-seq with rRNA depletion to identify 1,363 lncRNAs from mixed tissues including spear leaf and mesocarp at six developmental stages in oil palm. In their study, 6,882 SNPs were detected within lncRNAs, 28 of which, belonging to 21 lncRNAs, were significantly associated with variations in fatty acid content (Xia et al., 2020).

sRNA is a post-transcriptional regulatory factor found in both prokaryotic and eukaryotic organisms, encompassing entities such as siRNA and miRNA. These sRNA modulate gene expression within cells by interacting with mRNA or proteins, thereby exerting crucial regulatory functions in various biological functions, including plant growth and development, signal transduction, and responses to biotic and abiotic stresses (Ormazábal et al., 2024). In prior study, A total of 79 miRNAs were identified in oil palm, comprising 52 known and 27 novel miRNAs. The known miRNAs corresponded to 229 distinct putative target genes within the oil palm genome. From these, miRNAs and their putative target genes that were differentially expressed under salinity stress were further selected for functional annotation analysis. The biological processes most frequently associated with these target genes were “regulation of transcription, DNA-templated” and “oxidation-reduction process.” At the molecular function level, “protein binding” and “DNA binding” represented the most enriched categories (Salgado et al., 2021). Salgado et al. (2022) conducted deep sequencing analysis of miRNAs in both normal and water-deprived oil palm trees subjected to a 14-day period of drought (Salgado et al., 2022). The results showed that 62 miRNAs exhibited significant differential expression under these stress conditions, with 5 miRNAs up-regulated and 57 down-regulated. Building on this work, our current study investigated miRNA expression in cold stressed oil palm. We discovered that miRNAs of 24 nucleotides in length were predominantly expressed. A comparative analysis revealed that a total of 638 miRNAs displayed differential expression between CK and cold-stressed plants.

Transcriptome analysis offers a swift and comprehensive insight into the molecular processes behind specific biological functions. Previous research has evaluated transcriptome profiling in F1 hybrid seedlings derived from crosses between the dura and pisifera subspecies of oil palm (Xia et al., 2020). Those studies identified a total of 51,452 DEGs, with 40,725 from the CK group and 49,500 from the cold-treated sample. In contrast, our study identified a large number of DEGs, totaling13,539. This discrepancy may be due to the distinct materials used in our study. GO analysis implied that the DEGs were predominantly contributed to cellular processes and metabolic processes, aligning with Lei et al.’s findings (2014) (Lei et al., 2014). Importantly, the entities modulated by DEMs and DELs were also prominent in these processes, implying their significant role in the response to cold stress. Additionally, KEGG pathway enrichment analysis revealed that the most significantly enriched pathway for DEGs were associated with metabolism, followed by the biosynthesis of secondary metabolites. DEMs and DELs were similarly found to be enriched in pathways related to metabolism, biosynthesis of secondary metabolites, carbon metabolism and plant hormone signal transduction. Taken together, these findings imply that these pathways play a crucial role in the adaptive response to cold stress. Based on transcriptome analysis, this study proposes a model of ncRNA-driven metabolic reprogramming to elucidate the adaptive response of oil palm to cold stress. The mechanism comprises two core components: i) GO and KEGG analyses indicated that differentially expressed genes (DEGs), along with targets of DEMs and DELs, were significantly enriched in metabolic processes, biosynthesis of secondary metabolites, carbon metabolism, and plant hormone signal transduction. Notably, key genes involved in phenylpropanoid metabolism (e.g., *PAL*) and lipid synthesis (e.g., *FabF1*, *SAD1*) were found to be targeted by multiple ncRNAs (Kim et al., 2020; Wei et al., 2024). These results suggest that lncRNAs and miRNAs reconfigure carbon allocation under cold stress by modulating these metabolic pathways, promoting the production of protective secondary metabolites (such as flavonoids and lignin) while repressing oil biosynthesis, thereby maintaining energy homeostasis and membrane integrity. ii) Enrichment of plant hormone signaling pathways (e.g., auxin and abscisic acid) further suggests that ncRNAs may indirectly regulate downstream metabolic gene expression through hormone-responsive factors (Luo et al., 2022). For example, cold-induced lncRNAs (e.g., MSTRG.29739.2) and miRNAs (e.g., miR156-z) target key lipid synthesis enzymes (including *FabF1* and *MCMT*), leading to post-transcriptional suppression of oil accumulation and concurrent promotion of phenylpropanoid biosynthesis. This shift enhances cellular antioxidant defense and cold tolerance. Overall, this model positions ncRNAs as high-level regulatory hubs that coordinate hormone signals and metabolic pathways to reprogram primary and secondary metabolism under cold stress, ultimately fine-tuning the cold adaptation response in oil palm.

LncRNAs exert their regulatory functions in two primary modalities: as cis-acting elements, which modulate gene expression in proximity to their own transcription sites through various mechanisms, and trans-acting elements, which can influence genome-wide expression or gene localization by interacting with distant transcription activators or repressors (trans function) (Zhu et al., 2013; Grammatikakis and Lal, 2022; Herman et al., 2022). MicroRNAs (miRNA) are known to negatively regulate the expression of their target genes (Huang et al., 2021). The intricate interplay among different RNA species is well-documented; however, there is a paucity of study on lncRNA-miRNA-mRNA regulatory networks specifically in the context of cold stress on oil palm. This study represents a systematic identification and analysis of DELs, DEMs, and DEGs between CK and cold-stressed oil palm plants, and the construction of their interaction networks, a first in this field. The network construction is pivotal for a deeper understanding and elucidation of the molecular mechanisms underlying cold resistance in oil palm. Our analysis pinpointed 5 pivotal lncRNAs, 2 miRNAs and 1 gene that could act as key regulatory factors in the response to cold stress. Notably, the hub gene *ncbi_105033424*, which encodes phenylalanine ammonia-lyase (PAL), was significantly up-regulated (log_2_ ratio CK/cold stress = 5.6). Chen et al. (2008) reported that heat treatment can enhance PAL activity and the transcription of MaPAL1 and MaPAL2, thereby mitigating cold damage in banana fruits. By analogy, the up-regulation of the PAL-encoding gene (*ncbi_105033424*) may augment cold tolerance in oil palm (Chen et al., 2008). Additionally, our study observed that the hub miRNA miR156-z was induced, while miR395-y was repressed under cold stress conditions. Zhou et al. (2019) reported that overexpression of miR156 in rice can increase cell viability and growth rate under cold stress in Arabidopsis (Zhou et al., 2019). Similarly, in *Populus simonii* × *P. nigra*, It was found that miR395 was down-regulated following cold treatment (Zhou et al., 2019). The modulation of miR156 and miR395 in oil palm trees may thus enhance the plant’s adaptability to temperature fluctuations. The 5 identified hub lncRNAs were found to be enriched in metabolic pathways and the biosynthesis of secondary metabolites, as per KEGG pathway analysis. However, the precise biological roles of these lncRNAs remain to be fully elucidated. The cold treatment duration in this study was relatively brief (only 8 hours). Future investigations should incorporate extended cold stress time points to more fully elucidate the dynamic response of oil palm to low temperatures. Sampling could also be expanded to include other organs likely involved in cold stress responses, such as roots and stems, to better characterize organ-specific regulatory mechanisms. As oil palm genetic transformation systems continue to mature, subsequent studies are encouraged to employ stable genetic transformation and *in vivo* functional assays to validate molecular interactions and strengthen mechanistic conclusions. Further research would benefit from a integrated approach combining multi-timepoint sampling, multi-tissue analysis, experimental verification of target genes, and stable transformation techniques to advance the understanding of cold tolerance in oil palm.

KEGG pathway enrichment analysis has demonstrated that DEGs as well as the targets of DEMs and DELs, are significantly enriched in metabolic pathways and biosynthesis of secondary metabolites. Palm oil, a prominent secondary metabolite of the oil palm, is predominantly synthesized within the plastids and endoplasmic reticulum (Xia et al., 2019). In the present study, we identified seven DEGs within the palm oil biosynthesis pathway and five DELs predicted to regulate these DEGs. Notably, four key genes, *MCMT*, *FabB3*, *FabA*, and *SAD1*, were found to be significantly down-regulated in oil palm. Additionally, five lncRNAs, predicted to target these four genes, also exhibited significantly suppressed expression in oil palm trees subjected to cold stress. Collectively, these findings suggest that palm oil biosynthesis is likely inhibited under cold stress conditions.

Protoplasts are plant cells with their cell walls removed, retaining complete genetic material and totipotency. They are widely used for gene function validation and regulatory pathway studies due to their high transformation efficiency and ability to express foreign genes transiently (Zhou et al., 2025). To further investigate the regulatory relationship between non coding RNA and target genes, a transient expression system of oil palm protoplasts was used to preliminarily identify and screen potential functional genes. The results showed that the expression of five predicted lncRNAs (*MSTRG. 29739.2*, *MSTRG. 29739.9*, *MSTRG. 4375.2*, *MSTRG. 41477.4*, and *MSTRG. 2307.2*) and four genes (*MCMT*, *FabB3*, *FabA*, and *SAD1*) did not show significant changes in protoplasts, and their regulatory relationship still needs further investigation. miR156-z was significantly upregulated, while *FabF1* was significantly down-regulated, indicating a negative regulatory relationship between the two genes. The interaction between miR156-z and *FabF1* may play a critical role in regulating lipid metabolism in oil palm under cold stress.

Oil palm, as a typical tropical economic crop, suffers from severely limited yield and geographic distribution due to cold stress. Elucidating its molecular mechanisms of cold tolerance is therefore essential for varietal improvement (Lee et al., 2024). Non-coding RNAs (ncRNAs), including miRNAs, lncRNAs, and circRNAs, have been established as critical “regulatory hubs” in plant stress responses (Cao et al., 2024; Chen et al., 2025). The following cutting-edge technologies provide systematic and in-depth approaches to decipher ncRNA functional networks:

- Degradome/PARE analysis enables precise identification of ncRNA target genes and their regulatory networks by validating miRNA-mediated cleavage and degradation of target mRNAs under stresses. This approach establishes a direct link between ncRNA regulation and functional phenotypes (Liu et al., 2022). By performing degradome sequencing on plant leaves and roots subjected to cold treatment, researchers can pinpoint miRNA-specific cleavage sites through detection of 5’ truncated mRNA fragments. Potential key targets may include genes central to cold tolerance, such as those regulating membrane lipid stability (e.g., fatty acid desaturases), ROS scavenging (e.g., antioxidant enzymes), signal transduction (e.g., calmodulins), and hormone pathways (e.g., ABA-responsive factors) (Fan et al., 2022). miRNA-mediated negative regulation allows precise spatiotemporal control of these pathways. Moreover, degradome data provide conclusive evidence that such regulation occurs post-transcriptionally (via mRNA stability) rather than at the transcriptional level.
- Lipidomics and fluxomics are employed to investigate how ncRNAs influence lipid and energy metabolism (key aspects of cold tolerance) via target gene regulation. Since cold tolerance correlates strongly with membrane lipid fluidity (determined by unsaturation degree) and integrity, comparing lipidomic profiles, particularly changes in phospholipid and glycolipid composition, fatty acid chain length, and unsaturation levels, among plant varieties with contrasting cold tolerance under cold stress can reveal metabolite-specific alterations (Barajas-Lopez et al., 2021). Integrating these data with expression patterns of upstream ncRNAs (e.g., miRNAs targeting fatty acid desaturase genes) and their target genes helps construct a complete regulatory axis: “ncRNA → target gene (enzyme) → lipid metabolite”.
- CRISPRi/a technology enables direct functional characterization of selected lncRNAs in regulating oil palm cold tolerance. CRISPR interference (CRISPRi) uses a catalytically inactive dCas9 fused to a repressor domain (e.g., KRAB) to target lncRNA promoters or transcripts, specifically knocking down expression without genomic DNA alteration. Conversely, CRISPR activation (CRISPRa) employs dCas9 fused to activators (e.g., VP64, p300) targeted to lncRNA promoters to induce overexpression (Omachi and Miner, 2022). Following CRISPRi/a manipulation in plant cell lines or protoplasts, cold stress assays can be conducted to evaluate phenotypes such as cell viability, membrane integrity (electrolyte leakage), and expression changes of relevant target genes (e.g., miRNA targets sponged by lncRNAs acting as ceRNAs).
- Stable overexpression or silencing of validated key ncRNAs in plant permits systematic evaluation of cold tolerance at the whole-plant level. Phenotypic assessments may include photochemical efficiency (Fv/Fm), leaf wilting/browning extent, recovery growth, and changes in lipidomic/physiological indicators. This whole-plant evaluation ultimately identifies transgenic lines with significantly enhanced cold tolerance, providing valuable germplasm for molecular breeding programs and representing a critical step toward practical application (Litholdo et al., 2018).

## 5 Conclusion

In this study, 1,106 DELs, 638 DEMs, and 13,539 DEGs were obtained through in depth analysis of whole transcriptome of oil palm under cold stress. The study demonstrated that miR156-z negatively regulates the expression of its target gene *FabF1* in oil palm protoplasts. These data provide a resource of ncRNA-centered regulatory candidates for cold response in oil palm. Targeted validation of the miR156-z–*FabF1* axis supports a role in lipid-metabolism adjustment; comprehensive functional assays (e.g., PARE, stable transgenics, lipidomics) will be critical to establish causality at the pathway level.

## Data Availability

The datasets presented in this study can be found in online repositories. The names of the repository/repositories and accession number(s) can be found in the article/**Supplementary Material**.

## Supplementary material

Supplementary Figure 1 Sequencing of the PCR-identified bacterial culture.

Supplementary Figure 2 Length distribution of miRNA reads.

Supplementary Figure 3 Gene Ontology enrichment analysis of DEGs.

Supplementary Figure 4 The top 20 enriched KEGG pathways of DEGs in CK and cold treated oil palm.

Supplementary Figure 5 GO enrichment analysis of the target genes of DEMs.

Supplementary Figure 6 GO enrichment analysis of the target genes of DELs.

Supplementary Figure 7 GO enrichment analysis of DEGs.
