# Supplementary material for: Regulatory roles of noncoding RNAs in oil palm response to cold stress
Source: Front Plant Sci. 2025 Oct 22;16:1685230. doi: 10.3389/fpls.2025.1685230 (PMC12587301; doi:10.3389/fpls.2025.1685230)
Supplement: Supplementary Figure 1 — Sequencing of the PCR-identified bacterial culture. [file DataSheet1.zip › Separate supplementary file.doc]

SUPPLEMENTARY FIGURE 1 Sequencing of the PCR-identified bacterial culture.

SUPPLEMENTARY FIGURE 2 Length distribution of miRNA reads.

SUPPLEMENTARY FIGURE 3 Gene Ontology enrichment analysis of DEGs.

SUPPLEMENTARY FIGURE 4 The top 20 enriched KEGG pathways of DEGs in CK and cold treated oil palm.

SUPPLEMENTARY FIGURE 5 GO enrichment analysis of the target genes of DEMs.

SUPPLEMENTARY FIGURE 6 GO enrichment analysis of the target genes of DELs.

SUPPLEMENTARY FIGURE 7 GO enrichment analysis of DEGs.
